# Prevalence and contributing factors of depression among women with infertility in low-resource settings: a systematic review and meta-analysis

**DOI:** 10.3389/fmed.2025.1477483

**Published:** 2025-02-27

**Authors:** Shimelis Tadesse, Henok Kumsa, Gemeda Wakgari Kitil, Alex Ayenew Chereka, Getnet Gedefaw, Fiker Chane, Esuyawkal Mislu

**Affiliations:** ^1^Department of Midwifery, College of Health Sciences, Mattu University, Mettu, Ethiopia; ^2^School of Midwifery, College of Health Science, Woldia University, Woldia, Ethiopia; ^3^Department of Health Informatics, College of Health Sciences, Mattu University, Mettu, Ethiopia; ^4^Department of Midwifery, College of Medicine and Health Science, Injibara University, Injibara, Ethiopia

**Keywords:** depression, depressive symptoms, infertility, women with infertility, systematic review and meta-analysis, Africa

## Abstract

**Background:**

Depressive symptoms are the most common manifestations of psychiatric disorders among women with infertility. In low-resource settings, the overall prevalence and contributing factors of depressive symptoms among women with infertility remain unknown.

**Objectives:**

To estimate the prevalence and contributing factors of depression among women with infertility in low-resource settings.

**Methods:**

A review was conducted using Preferred Reporting Items for Systematic Reviews and Meta-Analyses (PRISMA). The PubMed, MEDLINE, Google Scholar, and Cochrane databases were used to identify eligible studies published up to 30 November 2024. Three authors independently extracted the data. Studies that reported depression among women with infertility were included in this review. The data were analyzed with STATA version 14, and a meta-analysis was conducted using a random-effects model. Publication bias and heterogeneity were assessed via Eager’s tests and I^2^. Subgroup and sensitivity analyses were performed to identify the potential source/s of heterogeneity. A *p*-value of 0.05 was declared as statistically significant. The findings were synthesized and presented using texts, tables, and forest plots with measures of effect and 95% confidence interval (CI).

**Results:**

Seventeen published cross-sectional studies that met the inclusion criteria with a total of 3,528 women with infertility were selected for this study. The pooled prevalence of depression among women with infertility was 48.77% (95% CI (35.86, 61.67). Good functioning family {OR 0.71 [95% CI (0.51, 0.97), I^2^: 0.00%]}, good husband support {OR 0.52 [95% CI (0.34, 0.79), I^2^: 0.00%]}, primary infertility {OR 2.55 [95% CI (1.36, 4.79), I^2^: 68.53%]}, history of divorce {OR 4.41 [95% CI (2.11, 9.24), I^2^: 0.00%]}, and duration of infertility lasting more than 10 years {OR 6.27 [95% CI (2.74, 14.34), I^2^: 15.26%]} were statistically significant.

**Conclusion:**

Depression was high among women with infertility in low-resource settings such as Africa compared to those in high-income countries, men, and pregnant mothers. Good functioning family, good husband support, primary infertility, history of divorce, and duration of infertility lasting more than 10 years were statistically associated. Therefore, African countries and the stakeholders in collaboration with mental health experts and gynecological care providers should address these problems in order to reduce or prevent depression among women with infertility.

**Systematic Review Registration:**

PROSPERO (ID: CRD42024516458).

## Introduction

Infertility is defined as not being able to get pregnant (conceive) after 1 year (or longer) of unprotected sex for women of age less than 35 years or after 6 months for women of age 35 years old or older (1, 2). It is a significant global public health problem, affecting approximately 186 million (3), with an estimated prevalence of 8–12% among couples (4–7). However, the prevalence of infertility is higher in low-resource settings, such as in low- and middle-income countries, where it is estimated to be 31.1% (8). According to a World Health Organization (WHO) report, infertility is associated with various forms of disability, including physical, emotional, functional, or social, which may arise from its causes, treatments, or societal consequences (5, 9). These impacts may include chronic pelvic pain, reproductive organ loss, sexual dysfunction, psychiatric disorders, stigma or marginalization, and dependency (5, 9, 10). Infertility can be caused by multiple factors including male and female reproductive issues, lifestyle factors, socioeconomic status, and infections (7).

In low-resource settings, such as Africa, infertility in women commonly occurs due to pelvic inflammatory disease (39.38%), tubal factors (39.17%), and abortion (36.41%) (11). The burden of infertility is the highest (17.7%) in women in the age group of 35–44 years compared to those below this age group (12). Infertility affects women in low-resource settings in many ways, creating challenges not only for the couple but also for the entire family, leading to social and psychological issues (13). Infertility can be classified into two types: primary infertility with an estimate of 0.6–3.4% and secondary infertility with an estimate of 8.7–32.6% (14). Mental health disorders such as depressive symptoms are the most common psychiatric disorders among women with infertility problems (5, 6, 15–18).

Depression is a common and serious mental health condition characterized by persistent feelings of sadness, hopelessness, and a loss of interest or pleasure in activities once enjoyed. It can significantly interfere with a person’s daily life, relationships, and ability to function (19). In order to prevent or reduce the prevalence of depressive symptoms in women with infertility problems, interventions such as counseling, support, and treatment are essential within fertility centers (15, 20). Moreover, raising awareness of the burden and risk factors associated with infertility is essential to facilitate the provision of psychological interventions (6). However, the extent of depression and its risk factors among women with infertility may vary across different populations due to differences in culture, beliefs, healthcare settings, and socioeconomic status of the population (21).

The line of evidence shows that various contributing factors including duration of infertility, education status, employment status, income level, and social and family support, as well as spiritual wellbeing, have a remarkable association with this problem (17). Women with infertility and those without children often face societal discrimination and stigmatization, which can lead to psychological disorders such as anxiety and depression (22–25).

Depressive symptoms have major consequences on the mental wellbeing of women with infertility (26). This is because, in many low-resource settings, a woman’s identity and value are closely tied to her ability to bear children, especially sons. As a result, infertility often leads to social stigma, discrimination, and marginalization (9, 26). Women may experience rejection from their families and communities, marital strain, or even abandonment and divorce (7, 9, 26), which can ultimately affect their overall health and quality of life (21).

Although primary study findings from different parts of Africa exhibited different results that range between 21.8 and 92.7% (27, 28), there is a lack of comprehensive report findings on this issue. This highlights the need for a comprehensive assessment of the prevalence and contributing factors of depressive symptoms in women with infertility. To address this gap, the current study aimed to conduct a systematic review and meta-analysis to provide an estimated prevalence of depressive symptoms and its contributing factors in women with infertility in Africa. This study highlights the burden of infertility in Africa, raises awareness of the associated problems, and can help formulate strategies to prevent infertility in women and improve their quality of life.

## Methods

### Search strategy

Various search engines, such as PubMed, MEDLINE, Google, Google Scholar, EMBASSE, and Hinari, were used in this study to acquire relevant data from studies published until 10 November 2024. The following Medical Subject Headings (MeSH) terms were used to search published studies: (((((((depression) OR (depressive symptom)) AND (infertility)) OR (infertile)) AND (prevalence)) OR (magnitude)) AND (risk factor)) OR (determinant). Additionally, the references of the identified articles were also assessed.

### Inclusion and exclusion criteria

All observational studies reporting the prevalence of depressive symptoms and/or associated factors among women with infertility problems in Africa and those reported in English were included in this study, whereas case reports, review articles, studies of mental illnesses, non-English articles, and studies whose full text were not found were excluded from this study.

Population, Intervention, Comparison, Outcomes, and Study design (PICOS) criteria were applied to determine the eligibility criteria of the studies included in this review.

### Participants/population

All women of reproductive age with infertility problems were included.

### Intervention(s) and exposure(s)

Sociodemographic characteristics, personal habits and life experience, and medical and psychological health-related characteristics were the exposures of interest.

### Comparator(s)/control

Women with better sociodemographic status, good personal habits and life experiences, and no medical and/or psychological health problems were treated as a comparator/control group.

### Outcomes

The main outcomes of this study were the prevalence and associated factors of depression in women with infertility (both primary and secondary infertility), which were assessed using standard tools. These standard tools were Zung’s questionnaire sample, the Patient health questionnaire (PHQ-9), the Copenhagen Multi-Centre Psychosocial Infertility-Fertility Problem Stress Scales, the Beck Depression Inventory questionnaire, the Hospital Anxiety and Depression Scale, the 20-item Center for Epidemiologic Studies for Depression Scale, and the 30-item General Health Questionnaire.

### Data extraction

**Figure 1:**
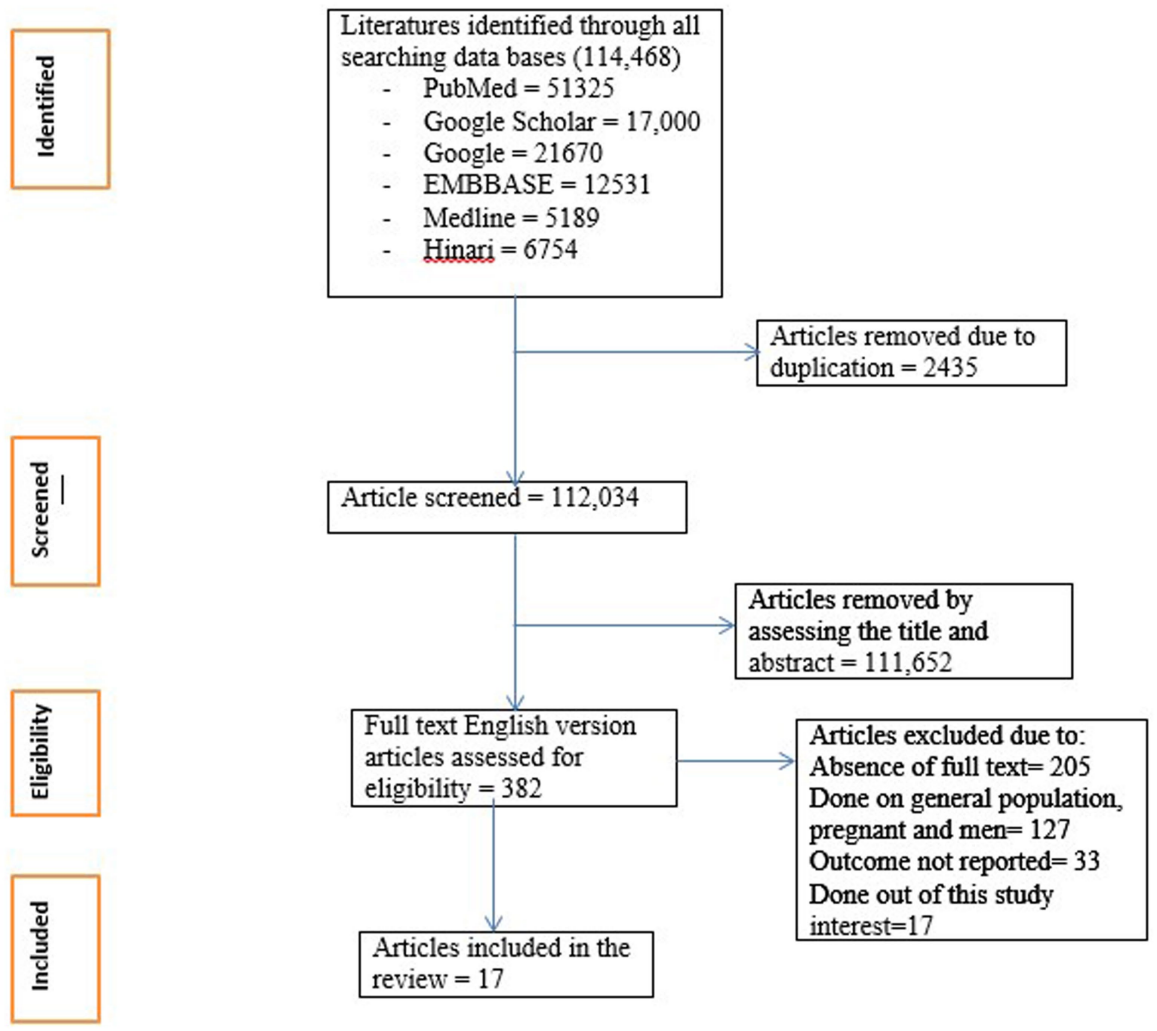
PRISMA flow diagram for article selection.

The data were extracted into Excel independently by three trained researchers. Then, the required data such as author names, year of publication, study settings, sample size, types of depression measurement tools, and prevalence and factors associated with depression were extracted (Supplementary Table 1). For studies with missing summary statistics, we reached out to the authors, when possible, to obtain the necessary data. If additional data could not be obtained, we applied statistical methods to estimate missing values. The PRISMA flow diagram was used to identify included studies (Figure 1).

### Data analysis

The quality of studies included in this review was assessed using the Joanna Briggs Institute (JBI) quality assessment tool (29, 30), and the Preferred Reporting Items for Systematic Reviews and Meta-Analyses (PRISMA) checklist was strictly followed throughout the study (31). The data were analyzed with STATA version 17. Publication bias was assessed via Egger’s test and with a funnel plot. Heterogeneity among included studies was assessed by computing the I^2^ tests. Subgroup and sensitivity analyses were performed to identify the potential source/s of heterogeneity. A random-effects model was used for variables with moderate to high heterogeneity, and a fixed-effects model was used for those with low heterogeneity. A *p*-value of 0.05 was declared to be statistically significant.

### Protocol

The protocol for this systematic review and meta-analysis was registered on PROSPERO (ID: CRD42024516458).

## Results

### Characteristics of included studies

A systematic review and meta-analysis was conducted on 17 published studies, with a total of 3,528 women with infertility, from four African countries (Figure 1). All the included studies were cross-sectional. Twelve studies were from Nigeria (32–43), two studies were from Ethiopia (28, 44), two studies were from Ghana (45, 46), and one study was from Uganda (47). Three studies were from East Africa (28, 44, 47), and fourteen studies were from West Africa (32–43, 45, 46).

### Prevalence of depression among women with infertility in Africa

**Figure 2:**
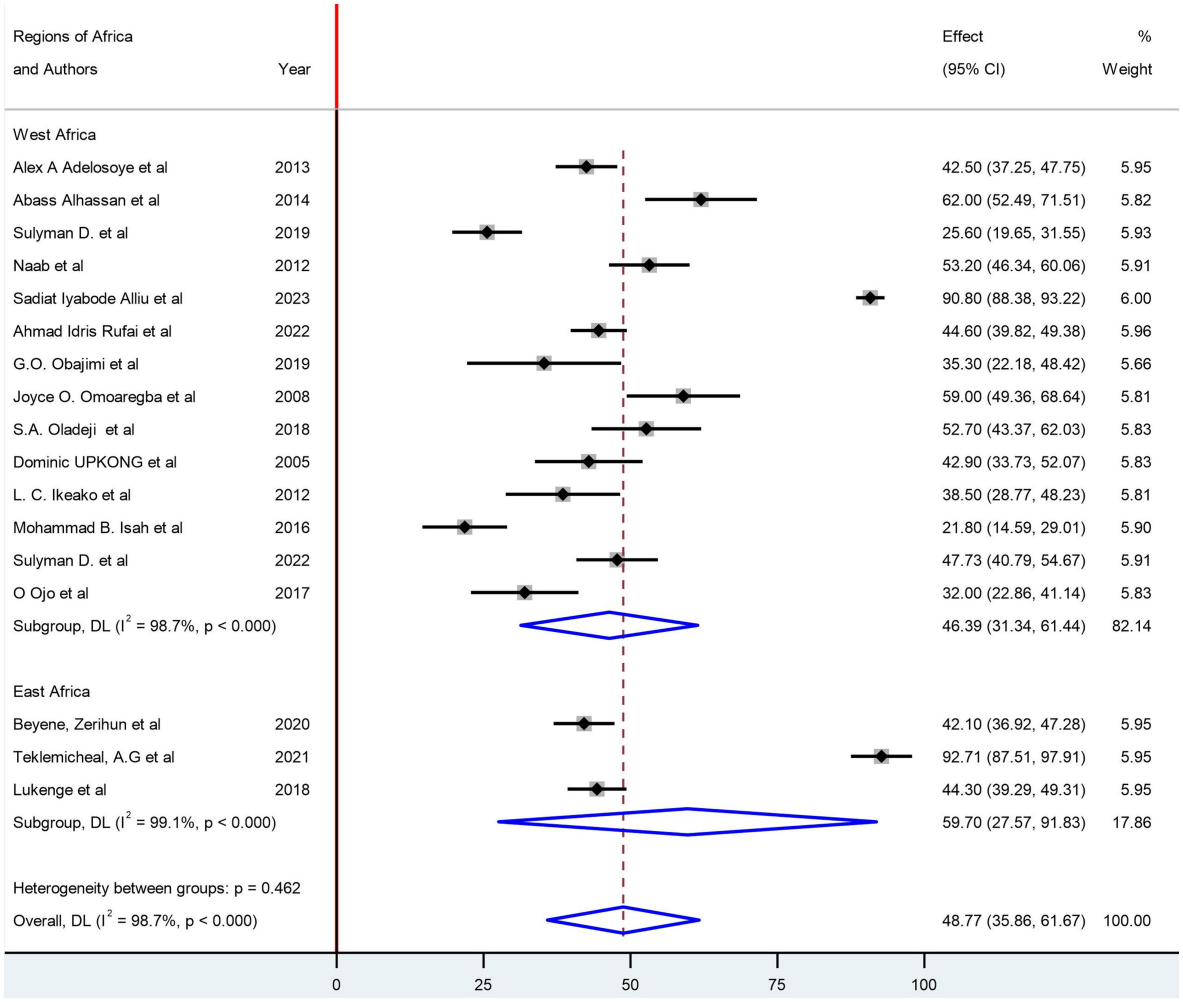
Subgroup analysis of the prevalence of depression based on the region of the country.

In this systematic review, publication bias and heterogeneity among studies were assessed. The graphical presentation via a funnel plot and Egger’s test (*p* = 1.00) did not identify the presence of possible publication bias (Supplementary Figure 1). However, there was a significant heterogeneity (I^2^: 98.7%) among the included studies (Figure 2).

**Figure 3:**
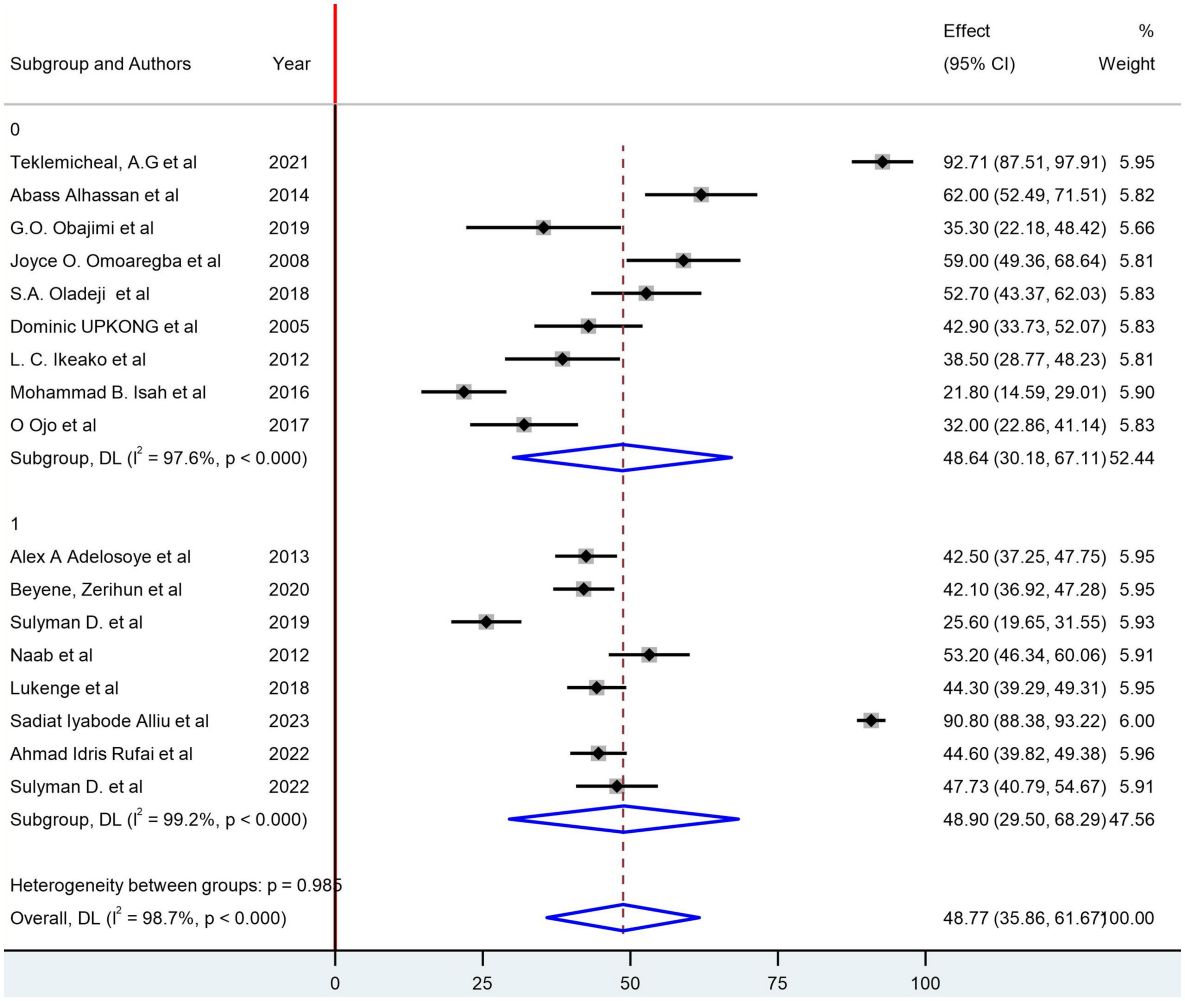
Subgroup analysis of the prevalence of depression based on the median sample size of included studies.

**Figure 4:**
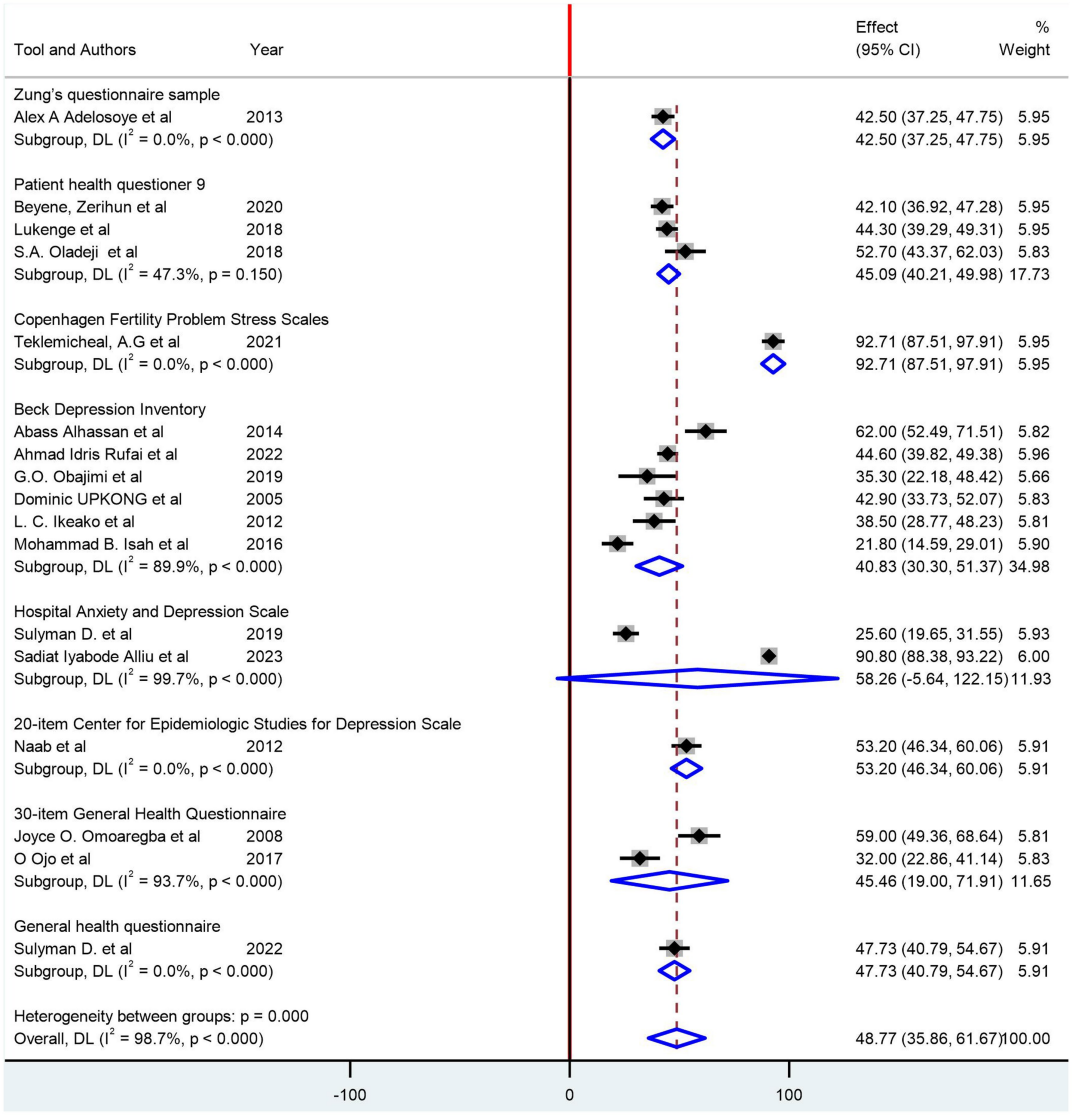
Subgroup analysis of the prevalence of depression among women with infertility based on the depression assessment tool.

The pooled prevalence of depression among women with infertility was identified as 48.77% (95% CI: 35.86, 61.67; I^2^ = 98.7%). In the subgroup analysis, based on the region of countries, the pooled prevalence of depression among women with infertility was 46.39% (95% CI: 31.34, 61.44; I^2^ = 98.7%) in West African countries and 59.70 (95% CI: 27.57, 91.83; I^2^ = 99.1%) in East African countries (Figure 3). Based on their sample size, studies with a sample size greater than the median value have a pooled prevalence of 48.90% (95% CI: 29.50, 68.29; I^2^ = 99.2%) and 48.64% (95% CI: 30.18, 67.11; I^2^ = 97.6%) (Figure 3). Additionally, a subgroup analysis based on the assessment tools was conducted (Figure 4).

### Factors associated with depression among women with infertility in Africa

#### Sociodemographic characteristics

**Figure 5:**
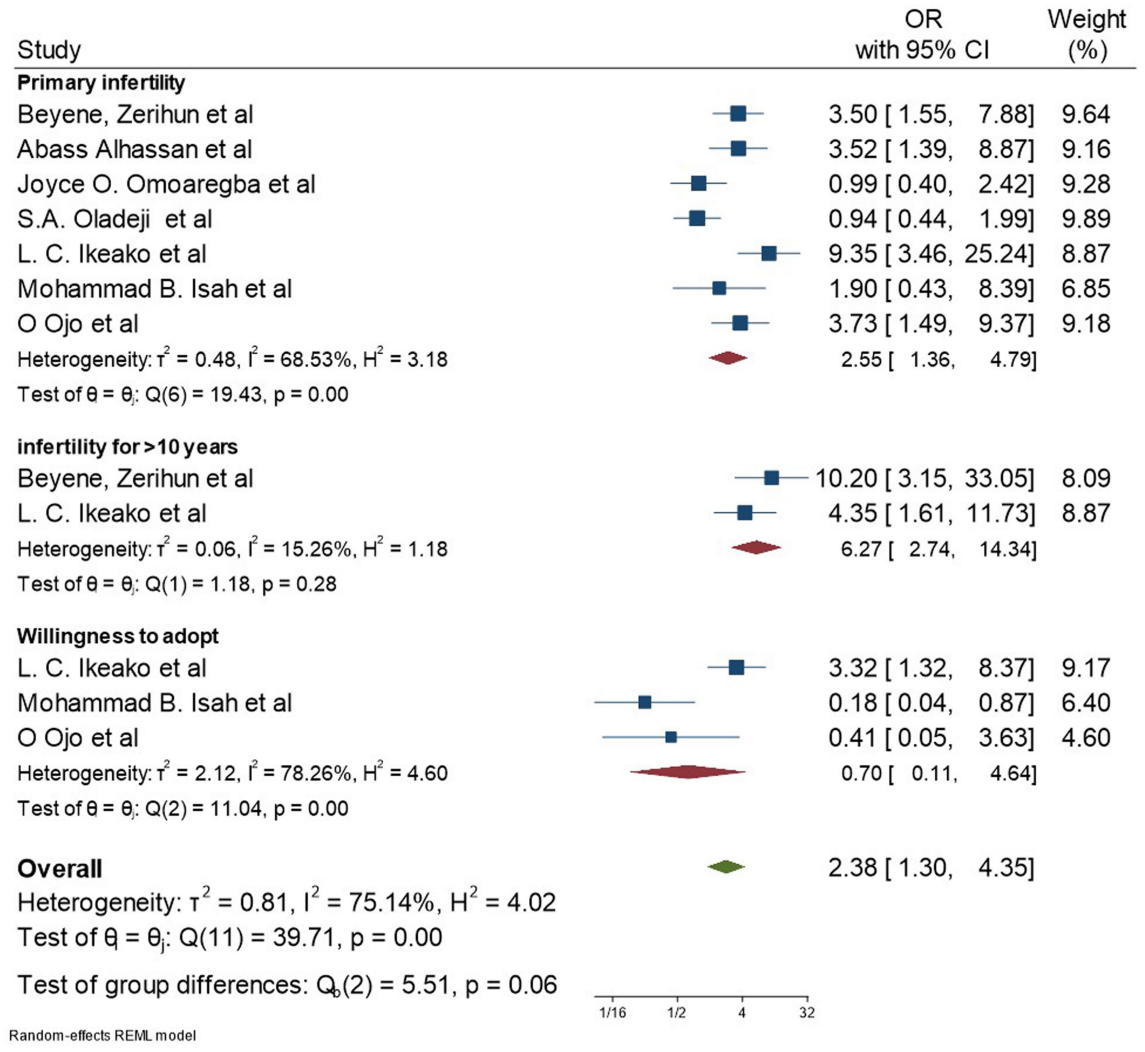
Forest plot for socio-cultural factors associated with depression among women with infertility.

Age (32, 34, 36, 37, 44), type of marriage (32, 34, 36, 39, 43, 45), history of divorce (43, 44), monthly family income (34, 44), educational level (34, 43), and religion (34) were identified from previous studies (Figure 5).

#### Sociocultural characteristics

**Figure 6:**
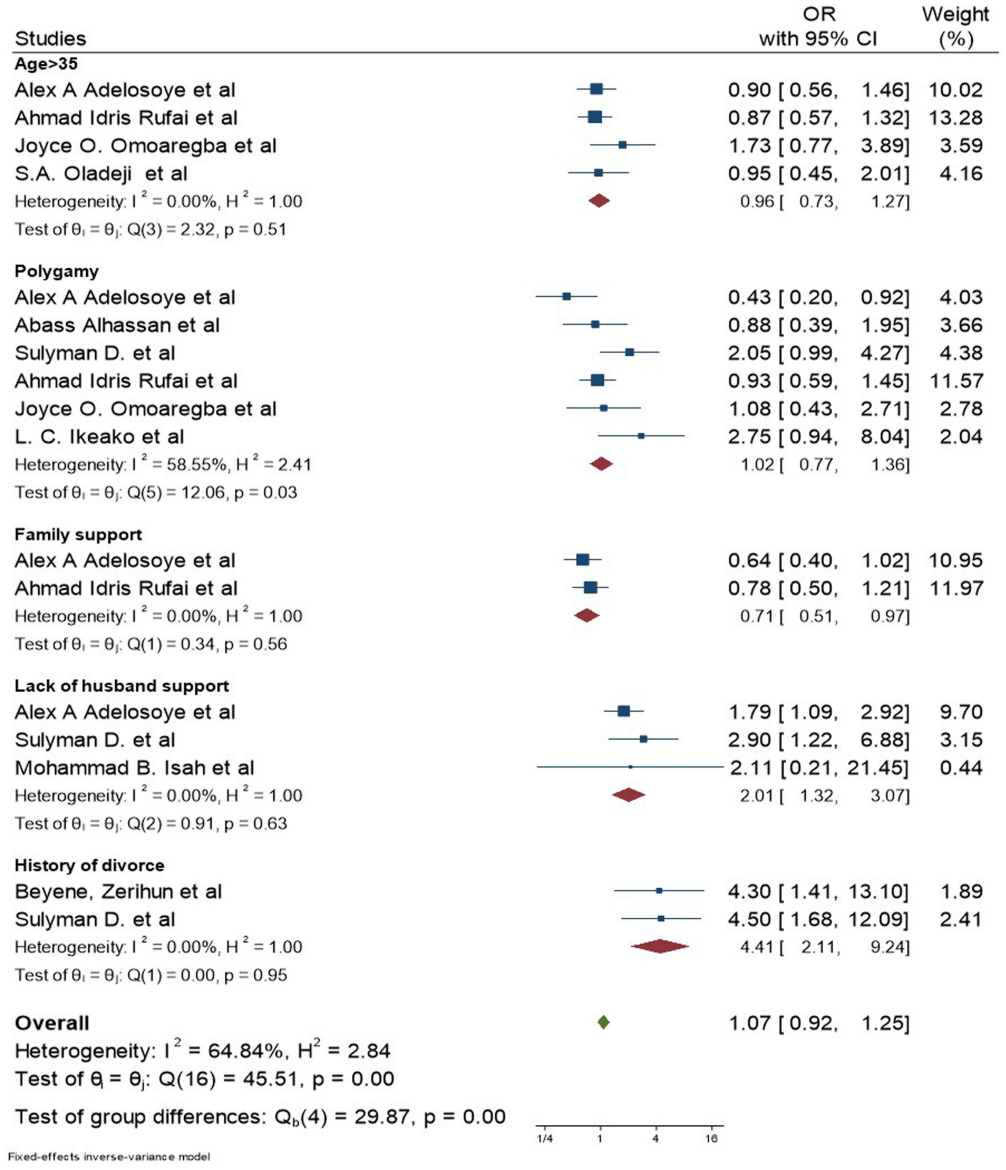
Forest plot for socio-demographic factors associated with depression among women with infertility.

Verbal assault by spouse and others (47), physical assault by spouse (47), sexual assault (47), stigmatizing behaviors (43), poor support from in-laws, difficulty in social function (37), willingness to adopt children (39, 40, 42), dysfunctional family support (32, 34), and dysfunctional husband support (32, 40, 43) were the sociocultural characteristics identified in the included studies (Figures 5, 6).

#### Infertility-related characteristics

Duration of infertility lasting more than 10 years (39, 44), primary infertility (36, 37, 39, 40, 42, 44, 45), surgical method of treatment (43), tubal factor as the cause of infertility (43), and a miscarriage history (47) were identified during data extraction. From the listed gynecological characteristics, the meta-analysis was performed only for the duration and type of infertility, as there was only a single study report for the other variables (Figures 5, 6).

After including the above-listed variables, this meta-analysis identified that depression among women with infertility was significantly associated with good functioning family (AOR: 0.71; 95% CI: 0.51, 0.97; I^2^: 0.00%), poor husband support (AOR: 2.01; 95% CI: 1.32, 3.07; I^2^: 0.00%), primary infertility 2.55 (95% CI: 1.36, 4.79; I^2^: 68.53%), history of divorce 4.41 (95% CI: 2.11, 9.24; I^2^: 0.00%), and duration of infertility lasting more than 10 years 6.27 (95% CI: 2.74, 14.34; I^2^: 15.26%). However, age greater than 35 years (AOR: 0.96; 95% CI: 0.73, 1.27; I^2^:0.00%), polygamous marriage (AOR: 1.02 95% CI: 0.77, 1.36; I^2^:58.55%), and women’s intention to adopt children (AOR: 0.70 95% CI: 0.11, 4.64; I^2^: 78.26%) were not significantly associated with depression among women with infertility. The funnel plot and Egger’s tests showed that there was no significant publication bias among the included studies (Supplementary Figures 2, 3).

The heterogeneity test (I^2^) among the variables significantly associated with depression revealed that, except for primary infertility (I^2^ = 68.53%), there was no significant heterogeneity for good functioning family, good husband support, history of divorce, or age greater than 35.

## Discussion

This study conducted a systematic review and meta-analysis to investigate the prevalence of depressive symptoms and the underlying factors associated with them in women struggling with infertility across Africa. The study found an overall pooled prevalence of depressive symptoms among women with infertility problems to be 48.77% (95% CI: 35.86, 61.67). The prevalence of depressive symptoms found in this study was higher than those among men with infertility problems (18.30%) (48) and pregnant mothers (20.7%) (49). The possible explanation is due to the fact that depressive symptoms are the most common disorder manifestations in women with infertility problems (16, 48), and this highlights the significant association between depressive symptoms and infertility problems (50). The prevalence of depressive symptoms found in the present study was also higher than that among high-income countries (28.03%) (21). A plausible explanation is due to the difference in sociodemographic characteristics, limited access to fertility and mental health treatment, cultural expectation and stigma, delay in seeking medical attention for their infertility due to lack of awareness, and under-resourced healthcare facilities to treat both infertility and depressive symptoms (21).

The present study identified the factors associated with depressive symptoms among women with infertility problems. These factors demonstrated that women who had a good functioning family were 29% less likely to have depressive symptoms than women who had a poor functioning family. This result was in line with a study conducted in Iran (51). A possible explanation for this is that women with a good functioning family will have proper behavioral control, roles, emotional responsiveness, and emotional involvement. These result in improving depressive symptoms among women with infertility problems (52).

In this study, women who had good husband support were 48% less likely to have depressive symptoms compared to their counterparts. This finding was supported by a study conducted in Japan that revealed that the lack of husband support was associated with depressive symptoms among women with infertility problems (53). A possible explanation is that women with infertility who get good husband support may have better decision-making practices about their health compared to their counterparts (53).

It was also revealed that women with primary infertility were 2.55 times more likely to have depressive symptoms than women with secondary infertility. This finding is supported by different studies conducted in Pakistan (54), Turkey (55), and Iraq (56). A possible reason may be that religious denial coping strategy was expected to be high in women with primary infertility, which resulted in the highest rate of depressive symptoms (57).

In this study, women who had a history of divorce were 4.41 times more likely to have depressive symptoms than women who had no history of divorce. This finding was supported by a study conducted in Iran. A possible reason could be because marital status is directly associated with happiness, and happiness is directly associated with mental health (58).

In this study, women with a duration of infertility lasting more than 10 years were 6.27 times more likely to have depressive symptoms than their counterparts. This finding is supported by different studies conducted in Iran (59), Iraq (56), and Turkey (60). This could be due to the fact that infertility for a prolonged period could reduce the possibility of treatment, resulting in higher depressive symptoms (60). This highlights the importance of early diagnosis and treatment of infertility to prevent or reduce depressive symptoms.

As a limitation, this review included only quantitative studies and studies that were published in the English language. Additionally, the cross-sectional nature of the studies does not indicate the true cause of the problem.

## Conclusion and recommendations

The results of this study indicated a higher prevalence of depressive symptoms in women with infertility problems in Africa than in high-income countries, men, and pregnant mothers. To prevent or reduce the problem, responsible organizations in Africa, in collaboration with mental health experts and gynecological care providers, should focus on improving proper family functioning and husband support, while giving special attention to women with primary infertility, a history of divorce, and infertility lasting more than 10 years. Therefore, African countries and the stakeholders can use this information to develop evidence-based strategies, policies, and health service delivery systems, as well as propose solutions to reduce or prevent depressive symptoms among women with infertility. It is also important for healthcare providers to consider depression when providing care for women with infertility and for future researchers to design interventional studies to address this problem.

## Data Availability

The original contributions presented in the study are included in the article/Supplementary material, further inquiries can be directed to the corresponding author.
