# Supplementary material for: Prevalence and contributing factors of depression among women with infertility in low-resource settings: a systematic review and meta-analysis
Source: Front Med (Lausanne). 2025 Feb 27;12:1477483. doi: 10.3389/fmed.2025.1477483 (PMC11903282; doi:10.3389/fmed.2025.1477483)
Supplement: Supplementary file 1 [file Supplementary_file_1.docx]

Supplementary Figures

Supplementary Figure 1. Funnel plot for studies included for depression among infertile women

Supplementary Figure 2. Funnel plot for sociodemographic and sociocultural factors for depression among infertility problems in low-resource setting

Supplementary Figure 3. Funnel plot for infertility related factors associated with depression among women with infertility problems in low-resource setting
