# Supplementary material for: Prevalence and contributing factors of depression among women with infertility in low-resource settings: a systematic review and meta-analysis
Source: Front Med (Lausanne). 2025 Feb 27;12:1477483. doi: 10.3389/fmed.2025.1477483 (PMC11903282; doi:10.3389/fmed.2025.1477483)
Supplement: Supplementary file 2 [file Supplementary_file_2.docx]

| List of Articles | 1. Were the criteria for inclusion in the sample clearly defined?* | 2. Were the study subjects and the setting described in detail?* | 3. Was the exposure measured in a valid and reliable way?* | 4. Were objective, standard criteria used for measurement of the condition?* | 5. Were confounding factors identified?* | 6. Were strategies to deal with confounding factors stated?* | 7. Were the outcomes measured in a valid and reliable way?* | 8. Was appropriate statistical analysis used?* | Overall appraisal score | Decision: Include(I) / Exclude(E) / Seek further info (SFI) |
| --- | --- | --- | --- | --- | --- | --- | --- | --- | --- | --- |
| Alex A Adelosoye et al | 1 | 1 | 1 | 1 | 3 | 3 | 1 | 1 | 6 |  |
| Beyene, Zerihun et al | 1 | 2 | 1 | 1 | 1 | 1 | 1 | 1 | 7 |  |
| Teklemicheal, A.G et al | 1 | 1 | 1 | 1 | 1 | 1 | 1 | 1 | 8 |  |
| Abass Alhassan et al | 1 | 1 | 1 | 1 | 1 | 1 | 1 | 1 | 8 |  |
| Sulyman D. et al (2019) | 1 | 1 | 1 | 1 | 2 | 1 | 1 | 1 | 7 |  |
| Naab et al | 1 | 1 | 1 | 1 | 2 | 2 | 1 | 1 | 6 |  |
| Lukenge et al | 1 | 1 | 1 | 1 | 1 | 1 | 1 | 1 | 8 |  |
| Sadiat Iyabode Alliu et al | 1 | 1 | 1 | 1 | 1 | 1 | 1 | 1 | 8 |  |
| Ahmad Idris Rufai et al | 1 | 1 | 1 | 1 | 1 | 1 | 1 | 1 | 8 |  |
| G.O. Obajimi et al | 1 | 1 | 1 | 1 | 2 | 2 | 1 | 1 | 6 |  |
| Joyce O. Omoaregba et al | 1 | 1 | 1 | 1 | 3 | 3 | 1 | 1 | 6 |  |
| S.A. Oladeji  et al | 1 | 1 | 1 | 1 | 1 | 1 | 1 | 1 | 8 |  |
| Dominic UPKONG et al | 1 | 1 | 1 | 1 | 1 | 3 | 1 | 1 | 7 |  |
| L. C. Ikeako et al | 1 | 1 | 1 | 1 | 2 | 2 | 1 | 1 | 6 |  |
| Mohammad B. Isah et al | 1 | 1 | 1 | 1 | 3 | 3 | 1 | 1 | 6 |  |
| Sulyman D. et al (2022) | 1 | 1 | 1 | 1 | 1 | 1 | 1 | 1 | 8 |  |
| O Ojo et al | 1 | 1 | 1 | 1 | 1 | 1 | 1 | 1 | 8 |  |
| *1: Yes; 2: No; 3: Unclear; N/A: not applicable | | | | | | | | | | |

JBI checklist
